# ACD15, ACD21, and SLN regulate the accumulation and mobility of MBD6 to silence genes and transposable elements

**DOI:** 10.1126/sciadv.adi9036

**Published:** 2023-11-15

**Authors:** Brandon A. Boone, Lucia Ichino, Shuya Wang, Jason Gardiner, Jaewon Yun, Yasaman Jami-Alahmadi, Jihui Sha, Cristy P. Mendoza, Bailey J. Steelman, Aliya van Aardenne, Sophia Kira-Lucas, Isabelle Trentchev, James A. Wohlschlegel, Steven E. Jacobsen

**Affiliations:** ^1^ Molecular Biology Institute, University of California Los Angeles, Los Angeles, CA 90095, USA.; ^2^ Department of Molecular, Cell and Developmental Biology, University of California Los Angeles, Los Angeles, CA 90095, USA.; ^3^ Department of Biological Chemistry, University of California Los Angeles, Los Angeles, CA 90095, USA.; ^4^ Eli and Edyth Broad Center of Regenerative Medicine and Stem Cell Research, University of California Los Angeles, Los Angeles, CA 90095, USA.; ^5^ Howard Hughes Medical Institute (HHMI), University of California Los Angeles, Los Angeles, CA 90095, USA.

## Abstract

DNA methylation mediates silencing of transposable elements and genes in part via recruitment of the Arabidopsis MBD5/6 complex, which contains the methyl-CpG binding domain (MBD) proteins MBD5 and MBD6, and the J-domain containing protein SILENZIO (SLN). Here, we characterize two additional complex members: α-crystalline domain (ACD) containing proteins ACD15 and ACD21. We show that they are necessary for gene silencing, bridge SLN to the complex, and promote higher-order multimerization of MBD5/6 complexes within heterochromatin. These complexes are also highly dynamic, with the mobility of MBD5/6 complexes regulated by the activity of SLN. Using a dCas9 system, we demonstrate that tethering the ACDs to an ectopic site outside of heterochromatin can drive a massive accumulation of MBD5/6 complexes into large nuclear bodies. These results demonstrate that ACD15 and ACD21 are critical components of the gene-silencing MBD5/6 complex and act to drive the formation of higher-order, dynamic assemblies at CG methylation (meCG) sites.

## Teaser

ACD15, ACD21, and SLN regulate accumulation of MBD6 to silence genes and TEs.

## INTRODUCTION

Eukaryotic organisms must properly localize macromolecules within cells to maintain homeostasis. Membrane-bound organelles serve this purpose, but recent discoveries have revealed the existence of membrane-less organelles or compartments (*1*–*3*). Often referred to as biological condensates, liquid-liquid phase-separated (LLPS) condensates, or supramolecular assemblies, these compartments such as stress granules, p-granules, heterochromatin, and the nucleolus concentrate proteins and nucleic acids to facilitate specific and efficient processes (*4*–*7*). If not properly controlled, the accumulation of these protein assemblies can lead to aggregates with detrimental impacts on cellular homeostasis and disease, yet how cells regulate these assemblies remains unclear (*3*, *8*, *9*). Molecular chaperones, such as heat shock proteins (HSPs), serve highly conserved roles to regulate the solubility, folding, and aggregation of proteins within cells, making them obvious candidates for the regulation of biological condensates (*10*–*16*). Small HSPs (sHSPs) use conserved α-crystalline domains (ACD) to form dimers which then facilitate large and dynamic oligomeric assemblies with client proteins, acting as a first line of defense against protein aggregation via a “holdase” activity (*14*). sHSPs further recruit J-domain–containing proteins (JDPs) which act as cochaperones for HSP70 proteins to maintain protein homeostasis (*14*, *17*–*19*). Both sHSPs and JDP/HSP70 pairs have been shown to associate with and regulate disease-related cellular condensates and assemblies across species (*20*–*23*).

In *Arabidopsis thaliana*, pericentromeric heterochromatin is condensed along with centromeres into nuclear bodies called chromocenters that contain most of the DNA methylated and constitutively silenced TEs and genes, as well as heterochromatic proteins such as DNA methylation binding complexes (*24*–*28*). Previous work has shown that multiple Arabidopsis DNA methylation binding complexes silence or promote the expression of genes and TEs through the recruitment of molecular chaperones with unknown functions (*29*–*31*). For example, methyl-CpG binding domain protein 5 (MBD5) and 6 (MBD6) redundantly silence a subset of TEs and promoter-methylated genes via recruitment of SILENZIO (SLN), a JDP. MBD5 and MBD6 also interact with two ACD-containing proteins called ACD15.5/RDS2 (AT1G76440.1) and ACD21.4/RDS1 (AT1G54850.1), hereafter referred to as ACD15 and ACD21 (*31*–*33*). While ACD15 and ACD21 have been implicated in silencing a transgene reporter, their specific chromatin functions remain unknown (*33*). Here, we demonstrate that ACD15 and ACD21 are necessary and sufficient for the accumulation of supramolecular, MBD5/6 complex assemblies at methylated CG (meCG) sites to silence genes and transposable elements (TEs). Moreover, they bridge the JDP SLN to MBD5/6 to regulate accumulation through the maintenance of mobility and dynamics of all complex components in these assemblies. We further demonstrate that MBD5/6 complex assemblies can be formed at discrete loci outside of chromocenters, in an ACD15- and ACD21-dependent manner, to cause gene silencing.

## RESULTS

### ACD15 and ACD21 colocalize with MBD5 and MBD6 genome-wide and are essential for silencing

**Fig. 1.**
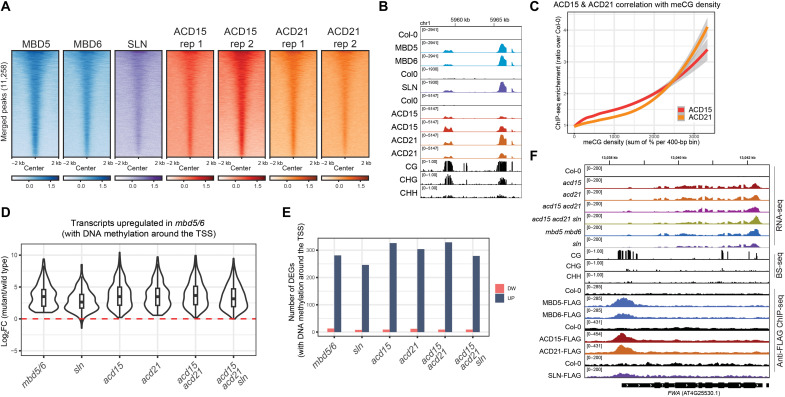
ACD15 and ACD21 are required for silencing. (**A**) Heatmap of FLAG-tagged MBD5, MBD6, SLN, ACD15, and ACD21 ChIP-seq enrichment (log_2_FC over no-FLAG Col-0 control) centered at all merged peaks. (**B**) Genome browser image of ChIP-seq data showing two methylated loci cobound by all MBD5/6 complex members. (**C**) Loess curves showing a correlation between ChIP-seq enrichment for a representative replicate and meCG density. (**D**) Violin plots showing mature pollen RNA-seq data for the indicated mutants, at *mbd5 mbd6* up-regulated transcripts (six replicates per genotype). (**E**) Comparison between genotypes of the number of RNA-seq differentially expressed genes (DEGs) with >40% meCG levels within a 600-bp window surrounding the TSS. (**F**) Genome browser image of ChIP-Seq and RNA-seq data at the *FWA* locus in the indicated genotypes. Wild-type (WT) bisulfite sequencing (BS-seq) data are shown as a reference. All RNA sequencing (RNA-seq) data are from pollen and ChIP-seq from flower buds.

We previously observed that MBD5, MBD6, and SLN pulled down two ACD-containing proteins named ACD15 and ACD21 (*31*). To investigate their binding patterns on chromatin, we performed chromatin immunoprecipitation sequencing (ChIP-seq) of FLAG-tagged ACD15 and ACD21. We observed that all five proteins colocalized genome-wide, and none of them appeared to have truly unique ChIP-seq peaks, suggesting that they could be recruited to DNA together as a complex (Fig. 1, A and B, and fig. S1A). Furthermore, ACD15 and ACD21 showed a nonlinear correlation with meCG density similar to MBD6 and SLN (*31*), suggesting that all MBD5/6 complex members accumulate preferentially at high-density meCG sites (Fig. 1C).

To test whether ACD15 and ACD21 are required for silencing, we generated *acd15* and *acd21* single mutants, an *acd15 acd21* double mutant, and an *acd15 acd21 sln* triple mutant via CRISPR-Cas9 (fig. S1B). RNA sequencing (RNA-seq) analysis revealed that all mutants showed very similar transcriptional derepression patterns at DNA methylated genes and TEs as compared to *mbd5 mbd6* and *sln* mutants (Fig. 1, D to F, and fig. S1, C and D). This includes the *FWA* gene which was previously shown to be silenced by the MBD5/6 complex (Fig. 1F) (*31*). While *acd15* and *acd21* mutants showed transcriptional derepression of genes and TEs, *acd15* and *acd21* mutants demonstrated no observable developmental phenotypes and produced normal progeny similar to *mbd5 mbd6* and *sln* mutants. These results demonstrate that ACD15 and ACD21 are critical components of the MBD5/6 complex required for silencing.

### ACD15 and ACD21 bridge SLN to MBD5 and MBD6

**Fig. 2.**
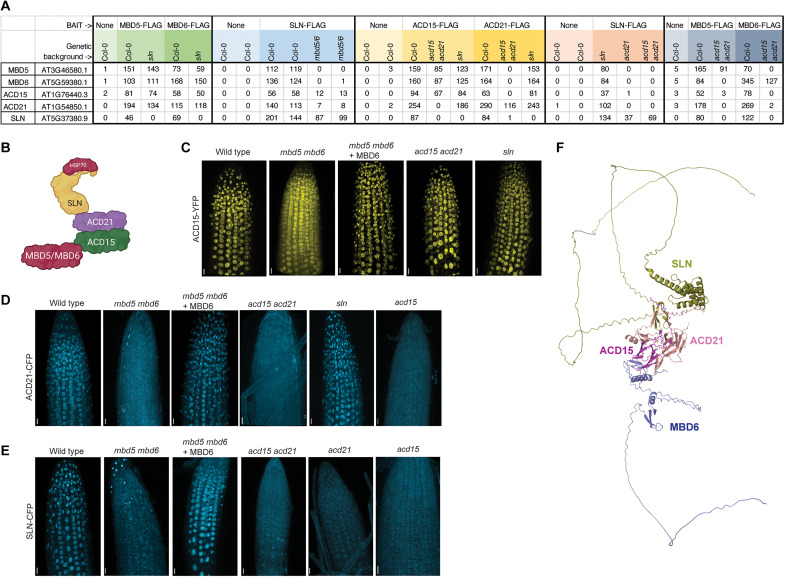
ACD15 and ACD21 bridge SLN to MBD5/6. (**A**) IP-MS of FLAG-tagged MBD5/6 complex members in the indicated genetic backgrounds (tandem mass spectrometry counts). (**B**) MBD5/6 complex organization as predicted by IP-MS. Created with BioRender.com (**C** to **E**) Three-dimensional reconstruction of root tips of plants expressing fluorescently tagged ACD15, ACD21, or SLN in WT (Col-0) and mutant backgrounds. Scale bars, 20 μm. (**F**) Predicted structure of MBD5/6 complex from AlphaFold-Multimer (*35*). Blue, MBD6; magenta, ACD15; maroon, ACD21; gold, SLN.

To determine the specific organization of the MBD5/6 complex, we performed immunoprecipitation–mass spectrometry (IP-MS) experiments using FLAG-tagged transgenic lines for each complex component in different mutant backgrounds (Fig. 2A and table S1). In the wild-type Col-0 background, ACD15 and ACD21 pulled down each other, MBD5, MBD6, SLN, and the same HSP70 proteins that were found to interact with the MBD5/6 complex previously (Fig. 2A and table S1) (*31*). MBD5 and MBD6 pulled down peptides of ACD15 and ACD21 in the absence of SLN, while SLN did not pull down MBD5 and MBD6 in the absence of ACD15 and ACD21, suggesting that ACD15 and ACD21 bridge the interaction between MBD5/6 and SLN (Fig. 2, A and B). Consistent with this model, ACD15 and ACD21 pulled down MBD5 and MBD6 in the *sln* mutant background, and SLN pulled down ACD15 and ACD21 in the *mbd5 mbd6* mutant background (Fig. 2A). ACD15 also pulled down MBD5 and MBD6 but not SLN in the absence of ACD21, while ACD21 did not pull down MBD5 and MBD6 in the absence of ACD15 (Fig. 2A). These results suggest that the MBD5/6 complex is organized such that MBD5 or MBD6 interact with ACD15, ACD15 interacts with ACD21, and ACD21 interacts with SLN (Fig. 2B).

To further study the organization and localization of MBD5/6 complex components, we used live confocal imaging of root tips to determine the cellular localization of fluorescent protein–tagged ACD15, ACD21, SLN, and MBD6. In wild-type plants, ACD15, ACD21, and SLN all showed clear nuclear localization which correlated strongly with nuclear MBD6 (Fig. 2, C to E, and fig. S2, A to C). ACD21, ACD15, and SLN all showed an increase in cytosolic signal in *mbd5 mbd6* mutant plants which was rescued by coexpression with MBD6, demonstrating that all members of the complex require interaction with genetically redundant MBD5 or MBD6 to maintain efficient nuclear localization (Fig. 2, C to E). The decrease in nuclear localization of SLN is also consistent with previous ChIP-seq experiments showing loss of chromatin-bound SLN in the absence of MBD5 and MBD6 (*31*). ACD15 maintained nuclear localization and correlation with MBD6 in *acd15 acd21* and *sln* mutant plants, whereas ACD21 lost nuclear localization and correlation with MBD6 in *acd15* and *acd15 acd21* mutants, but not in the *sln* mutant (Fig. 2, C and D, and fig. S2, D to G). Last, SLN nuclear localization and correlation with MBD6 decreased in *acd15*, *acd21*, and *acd15 acd21* mutant plants (Fig. 2E and fig. S2, H and I). ACD21, ACD15, and SLN protein expression and nuclear intensities did not change in *mbd5 mbd6*, *acd15 acd21*, or *sln* mutant plants suggesting that changes in cellular localization are not explained by protein degradation (fig. S3, A to F). These results demonstrate that ACD21 requires ACD15 for proper nuclear localization, while SLN requires both ACD15 and ACD21, consistent with the complex organization model suggested by IP-MS experiments (Fig. 2B).

We next used the protein folding algorithm AlphaFold-Multimer to predict protein-protein interactions within the MBD5/6 complex (*34*, *35*). AlphaFold-Multimer confidently predicted that ACD15 interacts with MBD6 (or MBD5), that ACD15 interacts with ACD21, and that ACD21 interacts with SLN, all consistent with our experimental data from IP-MS and confocal microscopy (Fig. 2F). When given two copies of each member of the complex (MBD6, ACD15, ACD21, and SLN), AlphaFold-Multimer also confidently predicted that ACD15 and ACD21 form a dimer of two heterodimers in the middle of the structure, suggesting that the MBD5/6 complex likely contains at least two copies of each protein (fig. S2, J to L). This is consistent with previous results showing dimer formation by ACD-containing sHSPs (*14*, *36*). Given the genetic redundancy of MBD5 and MBD6, the complex would be predicted to contain a minimum of two MBD5s, two MBD6s, or one MBD5 plus one MBD6 (fig. S2L). In line with this prediction, we found that MBD5 and MBD6 pull down each other in IP-MS data in wild type, but not in the *acd15 acd21* double-mutant background, indicating that ACD15/ACD21 facilitate interaction between two MBD5/6 proteins (Fig. 2A and fig. S2L).

### ACD15, ACD21, and SLN regulate heterochromatic localization, accumulation, and dynamics of the MBD5/6 complex

**Fig. 3.**
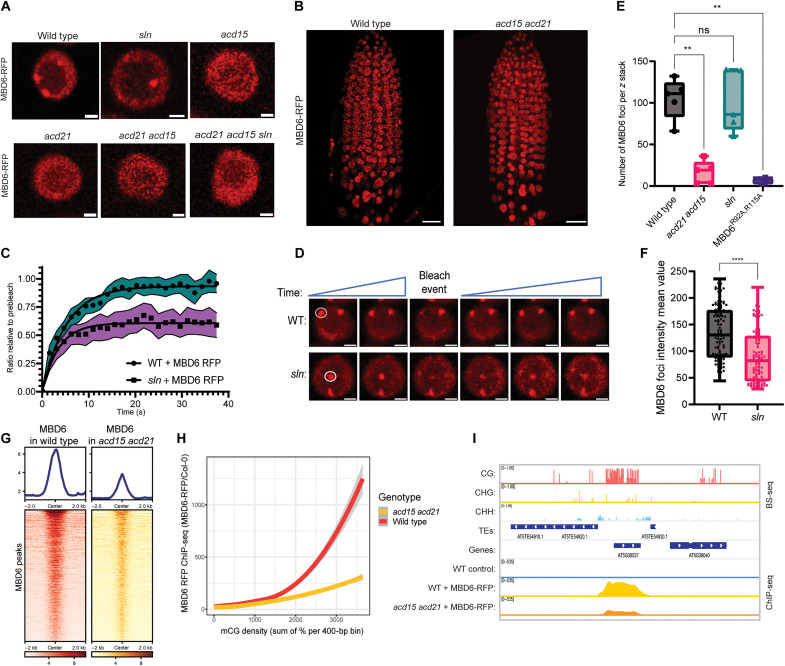
ACD15, ACD21, and SLN regulate MBD6 accumulation and mobility. (**A**) Representative MBD6-RFP nuclear images in mutant backgrounds. Scale bars, 2 μM. (**B**) Three-dimensional reconstruction of MBD6-RFP root tip *z* stacks. Scale bars, 20 μM. (**C**) Fluorescence recovery after photobleaching (FRAP) recovery curves comparing MBD6 signal in WT and *sln* plants. Shaded area: 95% confidence interval of FRAP data (*N* = 25 from five plant lines); dots: mean values, line: fitted one-phase, nonlinear regression. (**D**) Representative image of FRAP experiment. White circles indicate foci chosen for bleaching. Scale bars, 2 μM. (**E**) MBD6 foci counts across 50 slice *z* stacks of root meristems from five plant lines per genotype. Welch’s analysis of variance (ANOVA) and Dunnett’s T3 multiple comparisons test (**P < 0.01, ns: *P* ≥ 0.05). (**F**) Box plots of mean intensity values of MBD6 foci (five individual plants per genotype). Two-tailed *t* test (****P < 0.0001, *N* = 100 per genotype). (**G**) Heatmaps and metaplots of MBD6-RFP ChIP-seq signal (log_2_ ratio over no-FLAG Col-0 control) at peaks called in the “MBD6-RFP in wild-type” dataset. (**H**) Loess curves showing a correlation between MBD6-RFP ChIP-seq enrichment and meCG density. (**I**) Genome browser tracks showing an example of a high-density meCG site bound by MBD6-RFP (ChIP-Seq). WT BS-seq data are shown as a reference. ns, not significant.

Given the known role of molecular chaperones in the regulation of protein complexes and aggregates (*16*), we hypothesized that ACD15, ACD21, and SLN may regulate the dynamics of MBD5/6 nuclear complexes. To test this, we measured the nuclear localization and mobility of MBD6 in root cells using live-cell, confocal, fluorescence microscopy. In wild-type and *mbd5 mbd6* mutant plants, MBD6 formed foci, which colocalized with ACD15, ACD21, and SLN foci (Fig. 3, A and B, and fig. S4A). MBD6 foci also overlapped with 4′,6-diamidino-2-phenylindole (DAPI)–staining chromocenters, as previously shown when MBD6 was overexpressed in leaf cells (fig. S4B) (*37*). To measure the mobility of the MBD6 protein, we used fluorescence recovery after photobleaching (FRAP) experiments (*38*). FRAP in wild-type plants revealed that MBD6 moves rapidly within nuclei with a FRAP recovery half time (*t*_1/2_) of ~3.60 s back into chromocenters after bleaching (Fig. 3, C and D, and fig. S4D).

We next tested whether MBD6 nuclear distribution or mobility was altered in *sln* mutants. Although MBD6 formed a similar number of nuclear foci in *sln* compared to wild-type plants, these foci showed somewhat reduced fluorescence intensity, suggesting that MBD6 was accumulating less efficiently within heterochromatin (Fig. 3, A, E, and F). FRAP of MBD6 in *sln* mutant plants revealed a marked reduction in the mobile fraction of MBD6 resulting in a lack of full recovery of the signal after bleaching (Fig. 3, C and D, and fig. S4D). Similar FRAP experiments on ACD15 and ACD21 nuclear foci showed that both were highly mobile in wild-type plants (*t*_1/2_ of 3.63 and 4.30 s, respectively) but also showed a decrease in the mobile protein fraction with failures to recover full signal in *sln* mutant plants (fig. S4, D to H) and a decrease in the fluorescence intensity of foci in *sln* compared to wild type (fig. S4, I and J). This altered mobility occurred even though gene expression, protein levels, and nuclear intensities of MBD6, ACD15, and ACD21 were not substantially different between wild-type and *sln* mutant plants (figs. S2, A to E, and S4, K and L). Therefore, SLN regulates the mobility and, to some extent, the accumulation of the MBD5/6 complex.

Given the IP-MS, microscopy, and structure prediction results showing that ACD15 and ACD21 bridge the interaction between MBD6 and SLN, we expected *acd15 acd21* mutants to alter the FRAP mobility of MBD6 in a manner similar to *sln* mutants. However, we found that the number of MBD6 foci was markedly lower in *acd15 acd21* mutant plants compared to wild-type plants, with only occasional MBD6 foci observed (Fig. 3, A, B, and E). MBD6 nuclear signal in *acd15 acd21* mutant plants was more diffusely distributed across nuclei compared to either wild-type or *sln* plants (Fig. 3A). A decreased number of MBD6 foci and a lack of overlap of these foci with DAPI-stained chromocenters was also observed in *acd15*, *acd21*, and *acd15 acd21 sln* mutant plants (Fig. 3A and fig. S4C). Therefore, ACD15 and ACD21 are necessary for MBD6 to efficiently accumulate into nuclear foci. This effect was specific to ACD15 and ACD21 since the loss of IDM3 (LIL), an ACD protein in the MBD7 complex (*32*), did not affect the number of MBD6 nuclear foci (fig. S4, M and N).

The MBD6^R92A,R115A^ mutant, which lacks the two conserved arginine residues required for MBD6 to specifically bind meCG in vitro and in vivo (*31*), resulted in a marked drop in MBD6 foci compared to wild type demonstrating that meCG binding is required for the formation of MBD6 foci (Fig. 3E). To determine whether loss of MBD6 foci in *acd15 acd21* mutant plants is also due to loss of binding to meCG sites or is due to lack of protein accumulation with MBD5/6 complexes directly bound to DNA, we performed ChIP-seq of MBD6 in *acd15 acd21* mutants. In wild-type plants, MBD6 localized to previously published MBD6 peaks (*31*) and showed a nonlinear correlation with meCG density, displaying strong enrichment at high-density, methylated regions (Fig. 3, G to I). However, MBD6 chromatin enrichment in *acd15 acd21* mutant plants, although not abolished, was decreased markedly and showed much less of a preference for high-density meCG sites (Fig. 3, G to I). Thus, while ACD15 and ACD21 are not necessary for MBD6 to bind meCG sites, they are needed for the accumulation of high levels of MBD6 at high-density meCG sites, which is consistent with the decrease of observable MBD6 foci in *acd15 acd21* mutants (Fig. 3, A and B). This ACD-driven multimerization of MBD5/6 likely forms complexes containing multiple methyl-binding domains that bind cooperatively at high-density meCG sites.

Together, these results demonstrate that ACD15 and ACD21 are required for the high-level accumulation of MBD5/6 complexes in chromocenters and at high-density meCG regions, while SLN regulates the mobility of these complexes to maintain the dynamic recycling of proteins.

### The StkyC domain of MBD6 is required for gene silencing and recruits ACD15 to the complex

**Fig. 4.**
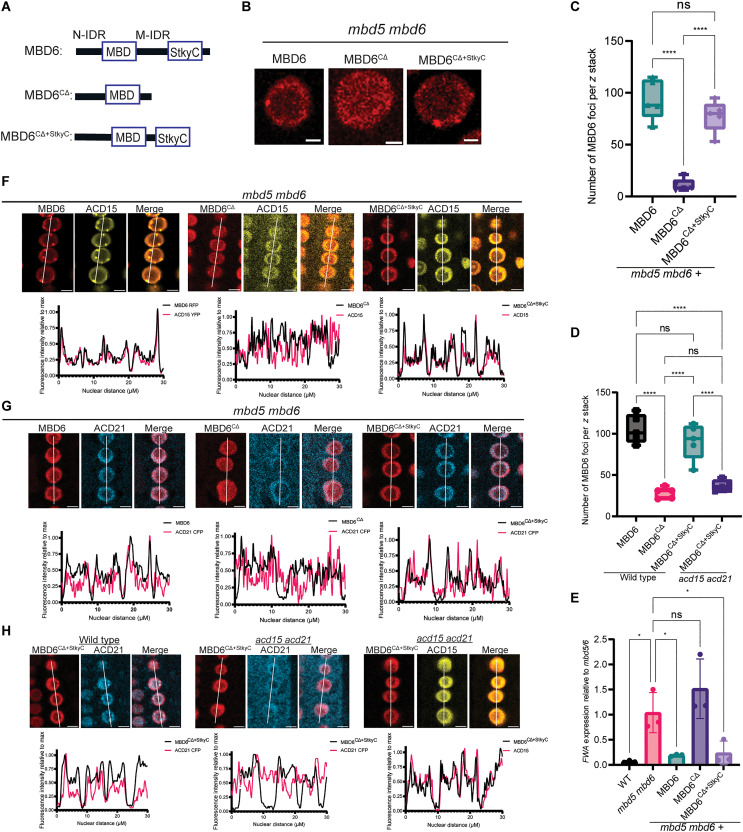
The StkyC domain of MBD6 is necessary for the function and localization of MBD6. (**A**) Graphical description of MBD6 mutant constructs. (**B**) Representative root nuclei showing MBD6-RFP signal. Scale bar, 2 μM. (**C** and **D**) Number of MBD6 nuclear foci (five different plant lines per sample, *z* stacks of 50 slices). Brown-Forsythe ANOVA with Tukey’s multiple corrections test (****P < 0.0001, ns: *P* ≥ 0.05). (**E**) *FWA* expression from reverse transcription quantitative polymerase chain reaction (RT-qPCR) of flower bud RNA. Brown-Forsythe ANOVA with Dunnett’s multiple corrections test (*P < 0.05, ns: *P* ≥ 0.05, *N* = 3 per genotype). (**F** to **H**) Representative images, along with nuclear profile plots, of MBD6-RFP with either ACD15-YFP or ACD21-CFP. White lines indicate the fluorescent regions, called nuclear distance, across multiple nuclei plotted in line graphs. Scale bars, 10 μM.

The AlphaFold-predicted structure of MBD6 reveals two structured domains, the MBD and a C-terminal domain of unknown function, as well as two intrinsically disordered regions (IDRs) (Fig. 4A and fig. S5A). The C-terminal folded domain shares amino acid similarity with the C terminus of two related MBD proteins, MBD5 and MBD7 (fig. S5B). This region of MBD7 has been termed the StkyC domain and is the proposed binding site for the ACD-containing IDM3 protein, which belongs to the same family as ACD15 and ACD21 (*30*). This suggests that the StkyC of MBD6 could interact with ACD15, and this interaction is confidently predicted by AlphaFold-Multimer (Fig. 2F and figs. S2, J and K, and S5A).

To experimentally determine what domains of MBD6 are necessary for gene silencing and chaperone interactions, we first truncated the N terminus [MBD6^NΔ^ (leaving amino acids 66 to 224)] or the C terminus of MBD6 [MBD6^CΔ^ (leaving amino acids 1 to 146)] (fig. S5C). To test whether these mutants are functional for silencing, we performed reverse transcription quantitative polymerase chain reaction (RT-qPCR) of the *FWA* gene, a target of the MBD5/6 complex (Fig. 1F) (*31*), in *mbd5 mbd6* mutant plants expressing full-length or truncated MBD6 alleles. *FWA* derepression in *mbd5 mbd6* plants was rescued by full-length MBD6-RFP (red fluorescent protein) or MBD6^NΔ^-RFP, but not by MBD6^CΔ^-RFP, showing that the middle IDR and/or the StkyC domain are required for MBD6 function (fig. S5D). MBD6^CΔ^ also showed a marked reduction in nuclear foci compared to full-length MBD6, a phenotype similar to that observed in *acd15 acd21* mutants and consistent with loss of the ACD15 binding site (Fig. 4, B and C, and fig. S5E).

To test whether the StkyC domain was critical, we added back the StkyC domain (amino acids 167 to 224) to MBD6^CΔ^ (MBD6^CΔ+StkyC^). MBD6^CΔ+StkyC^ was able to rescue MBD6 nuclear foci counts and complemented the derepression of *FWA* in *mbd5 mbd6* mutant plants (Fig. 4, B to E). MBD6^CΔ+StkyC^ expressed in *acd15 acd21* mutant plants formed very few nuclear foci, similar to the low number of MBD6^CΔ^ foci in wild-type plants, demonstrating that rescue of foci formation by the StkyC domain requires ACD15 and ACD21 (Fig. 4D). Notably, MBD6^CΔ^ and MBD6^CΔ+StkyC^ showed no substantial difference in gene expression or nuclear fluorescence intensity compared to full-length MBD6, suggesting that nuclear phenotypes are not caused by differences in the amount of MBD6-RFP within nuclei (fig. S5, F and G).

To determine whether the StkyC domain is responsible for localizing ACD15 and ACD21 to the MBD5/6 complex, we performed fluorescent protein colocalization experiments by coexpressing ACD21-CFP (cyan fluorescent protein) or ACD15-YFP (yellow fluorescent protein) with MBD6-RFP, MBD6^CΔ^-RFP, or MBD6^CΔ+StkyC^ RFP in *mbd5 mbd6* mutants. ACD15 and ACD21 both strongly correlated with full-length MBD6 [Pearson’s correlation coefficient (*r*) of 0.96 and 0.86, respectively] and overlapped well with MBD6 signal across root nuclei, whereas ACD15 and ACD21 showed much weaker correlations with MBD6^CΔ^ (*r* = 0.67 and 0.46, respectively) and lost overlap with MBD6^CΔ^ nuclear signal (Fig. 4, F and G and S5H-I). ACD15 and ACD21 also showed visibly higher cytosolic signal and lower nuclear signal when coexpressed with MBD6^CΔ^ in *mbd5 mbd6* mutant plants (Fig. 4, F and G and S5H-I). The addition of the StkyC domain (MBD6^CΔ+StkyC^) restored the correlation of ACD15 and ACD21 with MBD6 (*r* = 0.94 and 0.84, respectively), restored the overlap of ACD15 and ACD21 with MBD6 nuclear signal, and reversed the cytosolic localization of both ACD15 and ACD21 (Fig. 4, F and G, and fig. S5, H and I).

To further test whether ACD15 is needed for ACD21 to associate with MBD6^CΔ+StkyC^, we colocalized ACD15 and ACD21 with MBD6^CΔ+StkyC^ in wild-type or *acd15 acd21* double-mutant plants (Fig. 4H). ACD21 showed a reduced correlation with MBD6^CΔ+StkyC^ in *acd15 acd21* plants compared to wild type (0.48 vs 0.79), a reduced correlation with MBD6 signal across nuclei, and a visible increase in ACD21 cytosolic localization, suggesting that ACD21 requires ACD15 to associate properly with MBD6^CΔ+StkyC^ (Fig. 4I and fig. S5, J and K). On the other hand, ACD15 correlated strongly with MBD6^CΔ+StkyC^ in *acd15 acd21* plants (*r* = 0.92), maintained a strong nuclear signal, and directly overlapped with nuclear MBD6, demonstrating that ACD15 does not require ACD21 for proper localization with MBD6^CΔ+StkyC^ (Fig. 4H and fig. S5L). These experiments demonstrate that the StkyC domain of MBD6 is required for the function of the MBD5/6 complex, is needed for proper localization of ACD15 and ACD21, and mediates the accumulation of MBD6 at heterochromatic foci through ACD15 and ACD21.

### ACD15 and ACD21 can mediate functional and targeted gene-silencing foci

**Fig. 5.**
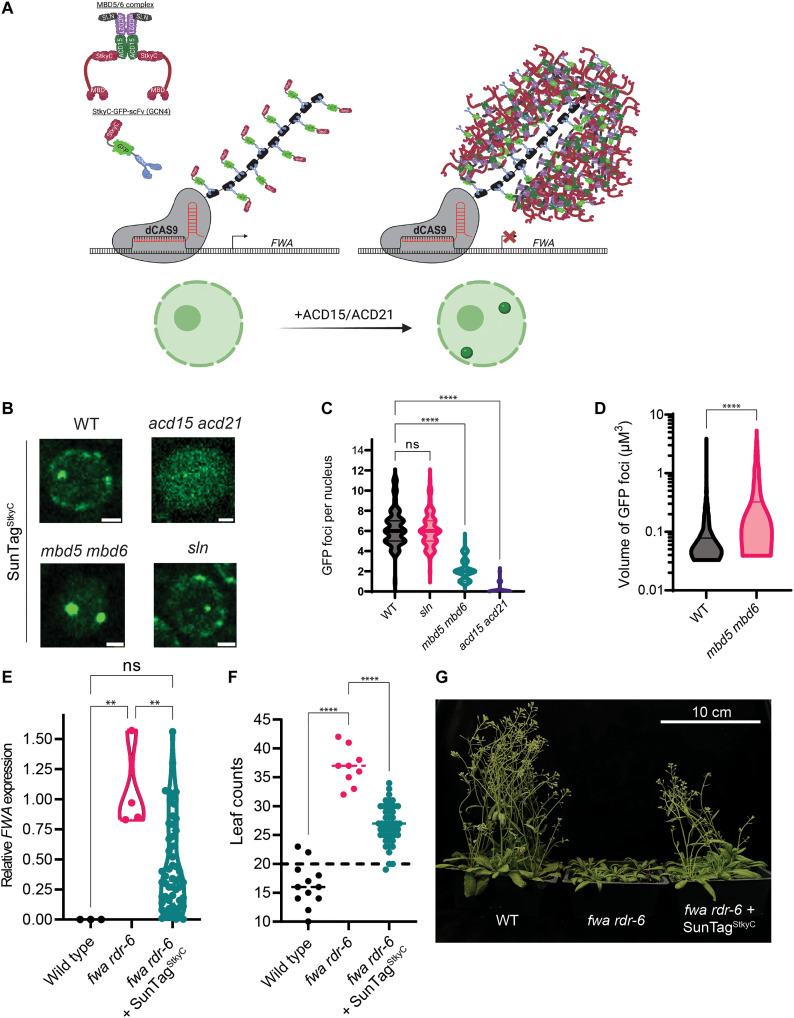
ACD15 and ACD21 drive the formation of MBD5/6 multimeric assemblies. (**A**) Graphical representation of SunTag^StkyC^ system and the hypothesized result. Created with BioRender.com. (**B**) Representative root nuclear images of SunTag^StkyC^ in different mutant backgrounds. Scale bar, 2 μM. (**C**) SunTag^StkyC^ GFP foci counts per nucleus (*N* = 100 per genotype). Compared using Welch’s ANOVA Dunnett’s T3 multiple comparisons test (****P < 0.0001, ns: *P* ≥ 0.05). (**D**) Volume of SunTag^StkyC^ GFP foci from five plant lines per genotype (WT: *n* = 1461, *mbd5 mbd6*: *N* = 1461 and 1371, respectively). Two-tailed *t* test (****P < 0.0001, ns: *P* ≥ 0.05). (**E**) RT-qPCR showing *FWA* expression in leaf tissue from T1 or control plants. Brown-Forsythe ANOVA with Tukey’s multiple corrections test (**P < 0.01, ns: *P* ≥ 0.05, *N* = 3, 4, and 55, respectively). (**F**) Leaf counts post flowering of T1 *fwa rdr-6* SunTag^StkyC^ plants. Brown-Forsythe ANOVA with Dunnett’s multiple comparisons test (****P < 0.0001, *N* = 12, 9, and 47, respectively). (**G**) Representative image of early flowering T2 *fwa rdr-6* plants expressing SunTag^StkyC^.

Some ACD-containing sHSPs are known to form dynamic oligomeric assemblies as part of their function in maintaining protein homeostasis (*18*), which could explain how ACD15/ACD21 drive high levels of MBD5/6 complex accumulation at meCG dense heterochromatin. To further explore this concept, we created a system to target MBD5/6 complexes to a discrete genomic location outside of pericentromeric heterochromatin. We utilized the SunTag system (*39*), composed of a dead Cas9 protein (dCas9) fused to 10 single-chain variable fragment (scFv) binding sites, targeted to the promoter of the euchromatic *FWA* gene (*40*). To nucleate MBD5/6 foci at the dCas9 binding site, we fused the scFv to the StkyC domain of MBD6 and to green fluorescent protein (GFP) to visualize the nuclear distribution of the fusion proteins (Fig. 5A).

If ACD15 and ACD21 drive higher-order multimerization of MBD5/6 complexes, then we would expect to observe discrete GFP foci in nuclei representing the dCas9 binding sites, as well as other foci corresponding to chromocenters since the scFv-GFP-StykC fusion would likely be recruited into multimerized MBD5/6 complexes within heterochromatin sites (Fig. 5A). We observed an average of 6.4 GFP foci per nucleus in SunTag^StkyC^-expressing wild-type plants (Fig. 5, B and C), some of which overlapped with DAPI staining chromocenters and others that did not (fig. S6A). We also transformed SunTag^StkyC^ into the *mbd5 mbd6* mutant, which would be predicted to eliminate recruitment of the scFv-GFP-StykC fusion protein into chromocenters by elimination of meCG bound endogenous MBD5/6 complexes. As predicted, we now observed an average of only two foci per nucleus (Fig. 5, B and C), likely corresponding to the *FWA* alleles on the two homologous chromosomes. Consistent with these foci representing dCas9 bound to euchromatic *FWA* (*40*), these foci did not overlap with DAPI staining chromocenters (fig. S6B). Notably, the volume of SunTag^StkyC^ foci was increased in *mbd5 mbd6* (Fig. 5D), with the vast majority of nuclear GFP signal accumulating at the two nuclear bodies (Fig. 5B), suggesting that excess scFv-GFP-StykC fusion protein shifted from heterochromatic regions to the dCas9 binding sites.

We also expressed the SunTag^StkyC^ system in *acd15 acd21* mutants to determine whether ACD15 and ACD21 are required for foci formation. SunTag^StkyC^ now only displayed diffuse nucleoplasmic GFP signal, lacking detectable foci (Fig. 5, B and C). This pattern was similar to control plants expressing a SunTag-TET1 system (*41*), in which the scFv was fused to GFP and the human TET1 protein, suggesting that the GFP foci observed in SunTag^StkyC^ are not a general property or artifact of the SunTag system (fig. S6C). We also introduced the SunTag^StkyC^ system into the *sln* mutant background and observed GFP foci counts and localization similar to the wild-type plants, showing around 6.1 foci per nucleus (Fig. 5, B and C). These results demonstrate that ACD15 and ACD21 are necessary and sufficient to drive the high-level accumulation of MBD5/6 complexes at discrete foci.

We next tested if the foci formed by the SunTag^StkyC^ system are capable of gene silencing. The *FWA* gene is normally methylated and silent in wild-type plants. However, stably unmethylated and expressed *fwa* epigenetic alleles exist that cause a late flowering phenotype (*42*, *43*). This allowed us to test whether the SunTag^StkyC^ system could silence *FWA* by introducing the system into the *fwa* epigenetic background. We found a significant suppression of *FWA* expression compared to *fwa* control plants (Fig. 5E). *fwa* plants expressing SunTag^StkyC^ also flowered earlier on average, showing a decrease in the number of leaves produced before flowering compared to *fwa* mutant plants (Fig. 5, F and G). The correlation of *fwa* expression with leaf counts from *fwa* SunTag^StkyC^ plants showed a strong positive correlation as expected (fig. S6D).

Last, we tested whether the SunTag^StkyC^ system could complement the *FWA* derepression phenotype of MBD5/6 complex mutants (Fig. 1E) (*31*). SunTag^StkyC^ was able to silence *FWA* in *mbd5 mbd6* mutant plants demonstrating that the tethering function of MBD6 could be largely replaced by targeting the StkyC domain and that silencing can occur without the methyl-binding proteins (fig. S6E). Unexpectedly, SunTag^StkyC^ could also partially complement *FWA* derepression in the *sln* mutant background, while SunTag^StkyC^ could not complement *FWA* derepression in the *acd15 acd21* mutant background suggesting that the formation of foci is directly linked to the silencing function (fig. S6, F and G). We further showed that protein levels of the scFv-GFP-StkyC were unchanged across wild type, *sln*, *mbd5 mbd6*, and *acd15 acd21* mutant plants demonstrating that correlations between foci formation and gene-silencing function by the SunTag^StkyC^ are not explained by a decrease in the amount of scFv-GFP-StkyC protein available to accumulate at *FWA* (fig. S6H). These results demonstrate that the SunTag^StkyC^ maintained some gene-silencing capability without SLN, suggesting that ACD15 and ACD21 alone have silencing ability.

## DISCUSSION

**Fig. 6.**
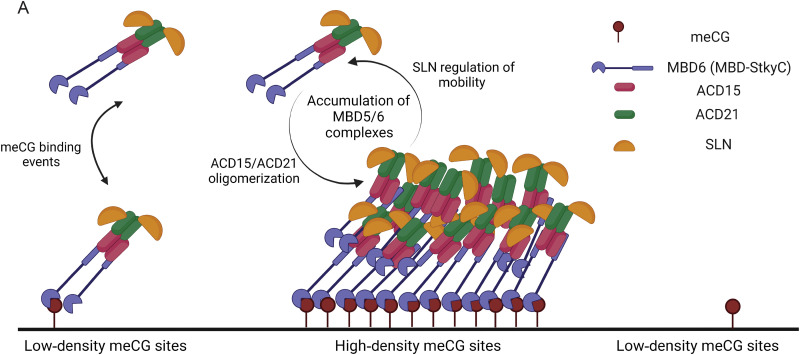
Model of MBD5/6 oligomerization at high-density meCG sites. (**A**) Diagram of proposed model showing ACD15/ACD21-dependent binding and accumulation of MBD5/6 complex members in multimeric assemblies. MBD5 and MBD6 recognize DNA methylation through their MBD domains to localize the complex to meCG sites. Although MBD5 or MBD6 can recognize individual meCG sites, regions with high-density meCG sites facilitate the recruitment of multiple MBD5/6 complexes, which triggers oligomerization: Once MBD5 and MBD6 are bound to DNA, ACD15 and ACD21 drive recruitment of other MBD5/6 complexes to facilitate oligomerization. This accumulation of proteins leads to higher-than-expected binding events and dwell time at meCG-dense regions. SLN directly interacts with ACD21, accumulates with the complex, and acts to maintain the mobility of proteins within the oligomeric assembly. Created with BioRender.com.

Our results provide evidence for distinct mechanistic roles for ACD15, ACD21, and SLN in the formation and regulation of the meCG-specific MBD5/6 silencing complex (Fig. 6A). ACD15 and ACD21 function to both drive the formation of higher-order MBD5/6 complex assemblies and bridge SLN to the complex. In contrast, the main role of SLN appears to be maintaining the dynamics of the complex by regulating the mobility of proteins within these assemblies. The activity of both ACD15/ACD21 and SLN are clearly required for the proper silencing function of the MBD5/6 complex. The accumulation of multiple MBD5/6 proteins into higher order complex assemblies can explain why these complexes preferentially localize to high-density meCG sites in the genome, likely via cooperative binding to closely spaced meCG sites. In addition, the loss of preferential MBD6 accumulation at high-density meCG sites in the *acd15 acd21* mutants suggests that ACD-driven multimerization is responsible for cooperative binding events.

ACD-containing sHSPs are found in all kingdoms of life and are most well known for their role in regulating the aggregation of proteins. In this capacity, other ACD-containing proteins are known to oligomerize to maintain proteostasis (*14*, *17*, *19*, *36*, *44*). In the MBD5/6 complex, however, the oligomerization capacities of ACD15 and ACD21 are specifically co-opted to control the multimerization and silencing function of the complex. Therefore, the MBD5/6 complex is a unique example of a DNA methylation binding complex which combines both the capacity to form supramolecular assemblies, along with recruitment of the regulatory protein (SLN) needed to maintain those assemblies in a fully dynamic form. SLN activity is dependent on its conserved histidine-proline-aspartic acid (HPD) tripeptide motif, which interacts with HSP70 chaperone proteins (*31*). HSP70s in turn are likely to use their disaggregation function to maintain the dynamics of proteins in the higher order MBD5/6 assemblies. Together, these activities result in the compartmentalization of functional silencing components to regions of high meCG density, contributing to efficient regulation of DNA methylated genes and TEs. It seems likely that ACD proteins in other systems may also play important roles outside of general protein homeostasis and may directly link supramolecular complex formation with cellular functions.

## MATERIALS AND METHODS

### Plant materials and growth conditions

All plants used in this study were in the Columbia-0 ecotype (Col-0) and were grown on soil in a greenhouse under long-day conditions (16-hour light/8-hour dark). Plants grown for microscopy were plated on ^1^/_2_ MS plates in growth rooms at room temperature (~25°C), with 16 hours of light and 8 hours of dark.

The following mutant lines were previously described: *mbd5 mbd6* transferred DNA (T-DNA) double mutant composed of *mbd5* T-DNA line SAILseq_750_A09.1 and *mbd6* T-DNA line SALK_043927 (*31*); *mbd5 mbd6* double mutant composed of *mbd5* CRISPR-Cas9–generated indel and *mbd6* T-DNA mutation SALK_043927 (*31*); and *sln* (SALK_090484) (*31*), *fwa rdr6-15* (*43*), and *lil-1* (*32*). Other mutants and transgenic lines were generated as described below.

### Generation of CRISPR lines

CRISPR-Cas9 mutants for ACD15.5 and ACD21.4 were generated with the pYAO::hSpCas9 system (*45*). We designed two guide RNAs per gene with the goal of generating large deletions, one of them targeting the beginning of the coding region and another one targeting the end of the gene (fig. S1B). We were not able to obtain large deletions at these loci, but we found small indels causing frameshifts (fig. S1B). The guide RNAs were cloned sequentially in the AtU6-26–sgRNA cassette by overlapping PCR. The PCR product was cloned into the SpeI site of the pYAO::hSpCas9 destination plasmid by In-Fusion (Takara, 639650). The procedure was repeated four times (two guides for each gene). The final vector was electroporated into AGLO agrobacteria and transformed into Col-0 or *sln* mutant plants (SALK_090484). T1 plants were selected on ½ MS agar plates with hygromycin B and were genotyped by PCR and by Sanger sequencing of PCR-amplified genomic regions surrounding each guide RNA. The lines containing the desired mutations were propagated to identify null segregants for the Cas9 transgene and to obtain homozygous mutations. Experiments were performed in the T4 generation.

### Generation of transgenic lines

The transgenic lines expressing FLAG-tagged constructs used for IP-MS and ChIP-seq were generated as follows. Genomic DNA was cloned into pENTR/D-TOPO vectors (Thermo Fisher Scientific), including endogenous promoters and introns, until the last base before the STOP codon. The MBD5 gene was cloned starting from 1094 base pairs (bp) before the transcription start site (TSS), MBD6 from 294 bp before the TSS, SLN from 2351 bp before the TSS, ACD15.5 from 644 bp before the TSS, and ACD21.4 from 266 bp before the TSS. The genes were then transferred via a Gateway LR Clonase II Enzyme mix (Invitrogen, 11791020) into a pEG302-based binary destination vector including a C-terminal 3xFLAG epitope tag. The final vectors were electroporated into AGL0 agrobacteria that were used for plant transformation by agrobacterium-mediated floral dipping. T1 transgenic plants were selected with hygromycin B on ½ MS agar medium or with Basta (glufosinate) on the soil. IP-MS and ChIP-seq experiments were done in the T2 or T3 generation.

Transgenic plants expressing fluorescently tagged proteins were created using the pGWB553 (www.addgene.org/74883/), pGWB540 (www.addgene.org/74874/), and pGWB543 (www.addgene.org/74877/). Specifically, MBD6, ACD15, ACD21, and SLN promoters and coding sequences were PCR-amplified from genomic DNA (as explained above) and cloned into pENTR vectors (table S2). These coding sequences were then inserted into final destination vectors using Gateway LR Clonase II Enzyme mix (catalog no. 11791020, Thermo Fisher Scientific). These final destination vectors were then electroporated into AGLO and transformed into Col-0, *mbd5 mbd6* (SALK_043927), *sln* (SALK_090484), *acd15 acd21*, *acd21*, *acd15*, and *acd15 acd21 sln* (SALK_090484) mutant plants. Positive T1 plants were selected on ½ MS agar plates with hygromycin B and confirmed by Western blots using fluorescent protein–specific antibodies. For YFP and CFP Western blots a GFP-HRP (horseradish peroxidase) antibody from Abcam (catalog no. ab190584) was used as a dilution of 1:10,000.

Transgenic plants expressing SunTag^StkyC^ were created from a previously published SunTag^TET1^ plasmid using the StkyC domain sequence (amino acids 173 to 225) of MBD6 (*41*, *45*). The SunTag^StkyC^ was targeted using two guides [guide 4 (ACGGAAAGATGTATGGGCTT) and guide 17 (AAAACTAGGCCATCCATGGA)] which were cloned as previously described (*46*–*47*). This plasmid was electroporated into AGLO and transformed into Col-0, *mbd5 mbd6* (SALK_043927), *acd15 acd21*, *acd21*, *acd15*, *sln* (SALK_090484), and *fwa rdr-6-15* (*43*). Positive selection of transgenic plants was done on ½ MS agar plates with hygromycin B after 5 days in the dark at 4°C, 8 hours in the light at room temperature, and another 5 days in the dark at room temperature. Western blots were used to confirm the expression of the scFv-GFP-StkyC using a hemagglutinin-HRP antibody at a dilution of 1:3000.

### Immunoprecipitation–mass spectrometry

IP-MS experiments were performed as previously described (*31*). Briefly, 8 to 10 g of inflorescences for each sample was used. Frozen tissue was ground with a TissueLyser and resuspended in IP buffer [50 mM tris-HCl (pH 8.0), 150 mM NaCl, 5 mM EDTA, 20% glycerol, 0.1% Tergitol, 0.5 mM dithiothreitol, and cOmplete EDTA-free Protease Inhibitor Cocktail (Roche)]. Samples were filtered with miracloth, disrupted with a Dounce homogenizer, and centrifuged for 10 min at 4°C at 20,000*g*. Supernatants were incubated with 200 μl of Anti-FLAG M2 Magnetic Beads (Sigma-Aldrich, M8823) for 2 hours rotating at 4°C. The beads were washed five times in IP buffer and eluted with 3X-FLAG peptides (250 μg/ml) in TE. The eluted protein complexes were precipitated overnight with 20% trichloroacetic acid (TCA).

### Digestion and desalting

The protein pellets were resuspended with 100 μl of digestion buffer [8 M urea and 0.1 M tris-HCl (pH 8.5)]. Then, the samples were reduced and alkylated via sequential 20-min incubations with 5 mM tris(2-carboxyethyl)phosphine (TCEP) and 10 mM iodoacetamide at room temperature in the dark while being mixed at 1200 rpm in an Eppendorf thermomixer. Twenty microliters of carboxylate-modified magnetic beads [CMMB; also widely known as SP3 (*48*)] was added to each sample. Ethanol was added to a concentration of 50% to induce protein binding to CMMB. CMMB were washed three times with 80% ethanol and then resuspended with 50 μl of 50 mM tetraethylammonium bromide (TEAB).

The protein was digested overnight with 0.1 μg of LysC (Promega) and 0.8 μg of trypsin (Thermo Fisher Scientific, 90057) at 37°C. Following digestion, 1.2 ml of 100% acetonitrile was added to each sample to increase the final acetonitrile concentration to more than 95% to induce peptide binding to CMMB. CMMB were then washed three times with 100% acetonitrile, and the peptide was eluted with 65 μl of 2% dimethyl sulfoxide. Eluted peptide samples were dried by vacuum centrifugation and reconstituted in 5% formic acid before analysis by liquid chromatography–tandem mass spectrometry (LC-MS/MS).

### LC-MS acquisition and analysis

Peptide samples were separated on a 75 μM ID, 25-cm C18 column packed with 1.9 μM C18 particles (Dr. Maisch GmbH) using a 140-min gradient of increasing acetonitrile concentration and injected into a Thermo Orbitrap Fusion Lumos Tribrid mass spectrometer. MS/MS spectra were acquired using a data-dependent acquisition mode.

MS/MS database searching was performed using MaxQuant (1.6.10.43) (*49*) against the *A. thaliana* reference proteome TAIR (Araport11 release).

### Chromatin immunoprecipitation sequencing

The anti-FLAG ChIP-seq experiments were performed as previously described (*31*). The RFP ChIP-seq experiments (Fig. 3) were done with the following variations: (i) After sonication and two rounds of centrifugation, 50 μl of ChromoTek RFP-Trap Magnetic Agarose beads (catalog no. rtma, Proteintech) was added to each sample for overnight incubation. (ii) For elution, 250 μl of elution buffer (SDS 1% and NaHCO3 0.1 M) was added, and samples were shaken for 15 min at room temperature. This step was repeated twice to reach 500 μl of final elution volume. Eluate (480 μl) was combined with 20 μl of 5 M NaCl and incubated in a thermomixer overnight at 65°C and 400 rpm for reverse cross-linking. The following steps were performed as previously described (*31*).

ChIP-seq libraries were prepared with the Ovation Ultralow System V2 1–16 kit (NuGEN, 0344NB-A01) following the manufacturer’s instructions, with 15 cycles of PCR. Final libraries were sequenced with the Illumina NovaSeq 6000 System.

### ChIP-seq analysis

Raw reads were filtered on the basis of quality score and trimmed to remove Illumina adapters using Trim Galore (Babraham Institute). Filtered reads were mapped to the Arabidopsis reference genome (TAIR10) with Bowtie2 (*50*) with default parameters. PCR duplicates were removed using MarkDuplicates.jar (Picard Tools suite, Broad Institute). Genome browser tracks for visualization purposes were generated using deepTools (v3.0.2) bamCoverage (*51*) with the options –normalizeUsing RPKM and –binSize 10. To obtain tracks normalized over the no-FLAG control, we used deepTools bamCompare (*51*) with the “log2” option.

The analysis of the correlation between ChIP-seq data and mCG density was performed as previously described (*31*), by calculating the sum of meCG percentages in 400-bp bins. The data were plotted using the R package *ggplot* with the option *geom*_smooth.

ChIP-seq peaks were called with MACS2 (v 2.1.0) (*52*) using a false discovery rate cutoff of 0.01. The FLAG- and RFP-associated hyperchippable regions, defined as peaks called in the anti-FLAG Col-0 or anti-RFP Col-0 controls, were subtracted from the peak sets of each sample. The peaks of individual replicates for ACD15 and ACD21 were merged with *homer mergePeaks* using the option -d given (*53*). Overlap analysis of different ChIP-seq peak sets was performed with *homer mergePeaks* using the options -d given and -venn (*53*).

### RT-qPCR

RNA samples for RT-qPCR experiments were purified using a Direct-zol RNA Miniprep kit (catalog no. R2052, Zymo Research) from unopened flower bud tissue or leaf tissue used in Fig. 5. cDNA samples were prepared using SuperScript IV VILO Master Mix (catalog no. 11760500, Invitrogen) from ~400 ng of RNA and qPCR was performed using Bio-Rad Syber Green Master Mix (catalog no. 1708882, Bio-Rad). Each qPCR experiment contained two technical replicates for each gene (either *FWA*, *RFP*, or IPP2 housekeeping control) (table S3). qPCR results were analyzed using Bio-Rad CFX Maestro Software. *FWA* expression was normalized to the expression of the reference gene *IPP2* and to the control samples as indicated in each plot (i.e., *mbd5 mbd6* mutants or *fwa rdr-6* mutant) using the ΔΔCq method. The data were graphed using GraphPad Prism software. Statistical analysis was done as described in the figure legends.

### RNA sequencing

RNA-seq was performed on mature pollen samples isolated as previously described (*54*), with six biological replicates per genotype, grown and processed in two batches (three replicates each). Briefly, 700 to 1000 μl of open flowers were harvested in 2-ml protein low-bind tubes (Eppendorf). Galbraith buffer [700 μl; 45 mM MgCl2,30 mMC6H5Na3O7.2H2O (trisodium citrate dihydrate), 20 mM Mops, 0.1% (v/v) Triton X-100 (pH 7)] supplemented with 70 mM 2-mercaptoethanol was added to the tube, and the flowers were vortexed for 3 min at max speed in the cold room, to release the pollen from the anthers. The extraction procedure was repeated two times, and the two aliquots of pollen in solution were combined. The suspension was filtered with an 80-μm nylon mesh into a 1.5-ml tube, and then spun down for 5 min at 500*g*. The supernatant was carefully removed and the pollen was flash-frozen with a metal bead. Frozen samples were disrupted with a tissue grinder and RNA extraction was performed with the Direct-zol RNA MiniPrep kit (Zymo Research), with in-column deoxyribonuclease digestion. Approximately 500 ng of RNA was used as input for library preparation using the TruSeq Stranded mRNA Library Prep Kit (Illumina), according to the manufacturer’s instructions. The final libraries were sequenced with the Illumina NovaSeq 6000 System.

### RNA sequencing analysis

RNA-seq reads were filtered on the basis of quality score and trimmed to remove Illumina adapters using Trim Galore (Babraham Institute). The filtered reads were mapped to the Arabidopsis reference genome (TAIR10) using STAR (*55*), allowing 5% of mismatches (-outFilterMismatchNoverReadLmax 0.05) and unique mapping (-outFilterMultimapNmax 1). MarkDuplicates from the Picard Tools suite was used to remove PCR duplicates. Coverage tracks for visualization in the genome browser were generated using deepTools 3.0.2 bamCoverage with the options –normalizeUsing RPKM and –binSize 10 (*51*).

To obtain gene counts, we used a set of reference pollen transcriptome annotations that we previously generated and are available from Github at https://github.com/clp90/mbd56_pollen (*54*) (version: 15 Jun 2023, c002f91126495e34f6fad8b66f5128c6f7ed365b). We used HTSeq (*56*) with the option –mode = union, to obtain counts for all transcripts (genes, TEs, and other undefined noncoding transcripts). The HTSeq gene counts were used to perform the differential gene expression analysis using the R package DEseq2 (*57*) with a cutoff for the significance of *P*_adj_ < 0.05 and |log_2_FC| > 1. Figures were generated using the R packages *ggplot* and *UpSetR*.

To determine the promoter meCG levels at each transcript (Fig. 1D), we first identified promoters as a 600-bp region surrounding the TSS. Then, we calculated average meCG percentages at promoters using the bedtools map (*58*) with the option “mean.” Our previously published Col-0 flower buds bisulfite sequencing dataset was used for this analysis and for the representative genome browser tracks: GSM5026060 and GSM5026061 (combined replicates) (*31*).

### Amino acid alignment

Amino acid alignments of MBD5, MBD6, and MBD7 were performed using the Clustal Omega multiple sequence alignment tool (www.ebi.ac.uk/Tools/msa/clustalo/). Amino acid sequences MBD5 (accession no. Q9SNC0), MBD6 (accession no. Q9LTJ1), and MBD7 (accession no. Q9FJF4) were obtained from the UniProt protein database. The alignment was run with default settings.

### AlphaFold-Multimer protein structure prediction

Protein structure predictions were run with the AlphaFold Colab notebook (AlphaFold.ipynb, https://colab.research.google.com/github/deepmind/alphafold/blob/main/notebooks/AlphaFold.ipynb#scrollTo=XUo6foMQxwS2) (*34*, *35*). The standard workflow was followed, and “run_relax” option was disabled. The .pdb output files were visualized with PyMOL (Delano Scientific, LLC.).

### Leaf counting

Leaf counting was performed as mentioned previously (*31*) where total numbers of rosette and cauline leaves were counted in the T1 generation of plants grown side by side under the same conditions.

### Confocal microscopy

All confocal microscopy experiments were performed using the LSM 980 confocal microscope. Unless otherwise stated, all experiments were performed using ×40 magnification and a water objective lens. For all experiments using multiple fluorescent tags, we manually gated the excitation and emission spectrum to limit any cross-reactivity of the samples.

Live plant samples were prepared as follows: 2-week-old seedlings were grown on ½ MS plates at room temperature, ~25°C, and then transferred using forceps onto 1-mm-thick glass slides (catalog no. 12-550-08, Thermo Fisher Scientific) containing deionized water (room temperature). Seedlings were oriented such that root tips were on the middle of the slide, while leaves were extending from the top of the slides. Coverslips (#1.5; catalog no. 12-544-EP, Thermo Fisher Scientific) were placed on top of the plant, gently, so as not to destroy or stress the seedling. Usually, one to four plants were placed on one slide for imaging.

### FRAP experiment and analysis

FRAP experiments were performed on an LSM 980 using ×40 magnification and a water objective lens. Images of a region of interest were obtained as a “snap” to circle a region of interest to be bleached. Then, an experiment was run such that five images were taken followed by a bleaching event using 100% laser excitation wavelength, dependent on the fluorescent protein being imaged, for 300 iterations. The signal was then tracked after bleaching for an indicated amount of time. FRAP analysis was performed using EasyFRAP online analysis software (https://easyfrap.vmnet.upatras.gr/). Briefly, three regions of interest were measured for each FRAP replicate: (i) a bleached region, (ii) a specific nucleus region containing the bleached foci, and (iii) a random region containing no signal in the root of the plant. The signals of these three regions across time were added into the given Excel template from EasyFRAP and uploaded for analysis along with other replicate files (*N* = 25 for each FRAP experiment). Plotted FRAP curves represent the full normalization of the data to account for any variations in bleaching depth among samples. FRAP data starting from the bleaching event are plotted using GraphPad Prism software with 95% confidence intervals calculated from normalized FRAP data of FRAP experiment replicates. One-phase association and nonlinear regressions were fitted to estimate and statistically compare maximum plateau and *t*_1/2_ for each FRAP experiment.

### Quantification of foci counts, volume, nuclear distributions, and nuclear intensities

All foci counts, volumes, and nuclear distribution plots were quantified using ImageJ, image analysis software. Foci counts and volume measurements were obtained using the three-dimensional (3D) object counter app in the ImageJ software analyzing 50-slice *z* stacks of root meristems across multiple plant lines. Using the 3D objects counter, we established a size threshold to exclude any foci less than 0.3 μm to avoid any nonspecific background signal that would be inconsistent with MBD6 nuclear foci, but we did not exclude 3D objects on the edges of the counted objects. The 3D objects counter app in ImageJ uses an arbitrary numbering system for each image to establish a fluorescence signal threshold to allow the software to recognize 3D objects in any given image. To maintain consistent thresholding, the same signal threshold number was used across all images within the same experiments allowing the threshold setting to be the same across foci counts. Also, the *z* stack analyzed contained the same micrometer depth through the root across all replicates and used the same imaging settings (i.e., magnification and laser intensity).

Nuclear distributions were obtained using the plot profile feature across a fixed line length in ImageJ after converting images to RBG format. White lines (30 μM) represent the region, across nuclei, we plotted in line graphs seen below representative images. The plot profile measured the fluorescence intensity of each protein (MBD6, ACD15, ACD21, or SLN) along the white line. These fluorescence intensities were then normalized to the maximum intensity measured for each replicate to normalize the data distribution to the value of 1 and then plotted against the length of the white line (30 μM).

Foci counts, volumes, and nuclear distribution intensity values were all plotted using GraphPad Prism software, and statistical analysis was performed using GraphPad Prism software as mentioned in figure legends.

Nuclear mean value intensities were calculated by measuring the intensity of MBD6 within nuclei at the widest diameter nuclear region across a minimum of five plant lines for each tested plant line or MBD6 deletion mutant. This was performed using ZEISS, ZEN Blue software. These values were then plotted in GraphPad Prism and statically compared using GraphPad Prism.
