# Supplementary material for: ACD15, ACD21, and SLN regulate the accumulation and mobility of MBD6 to silence genes and transposable elements
Source: Sci Adv. 2023 Nov 15;9(46):eadi9036. doi: 10.1126/sciadv.adi9036 (PMC10651127; doi:10.1126/sciadv.adi9036)
Supplement: Supplementary file 1 — Figs. S1 to S6 Legend for table S1 Tables S2 and S3 [file sciadv.adi9036_sm.pdf]

Science Advances
AAAS

Supplementary Materials for

ACD15, ACD21, and SLN regulate the accumulation and mobility of MBD6 to silence genes and transposable elements

Brandon A. Boone *et al.*

Corresponding author: Steven E. Jacobsen, jacobsen@ucla.edu

Sci. Adv. **9**, eadi9036 (2023)
DOI: 10.1126/sciadv.adi9036

The PDF file includes:

Figs. S1 to S6
Legend for table S1
Tables S2 and S3

Other Supplementary Material for this manuscript includes the following:

Table S1

A

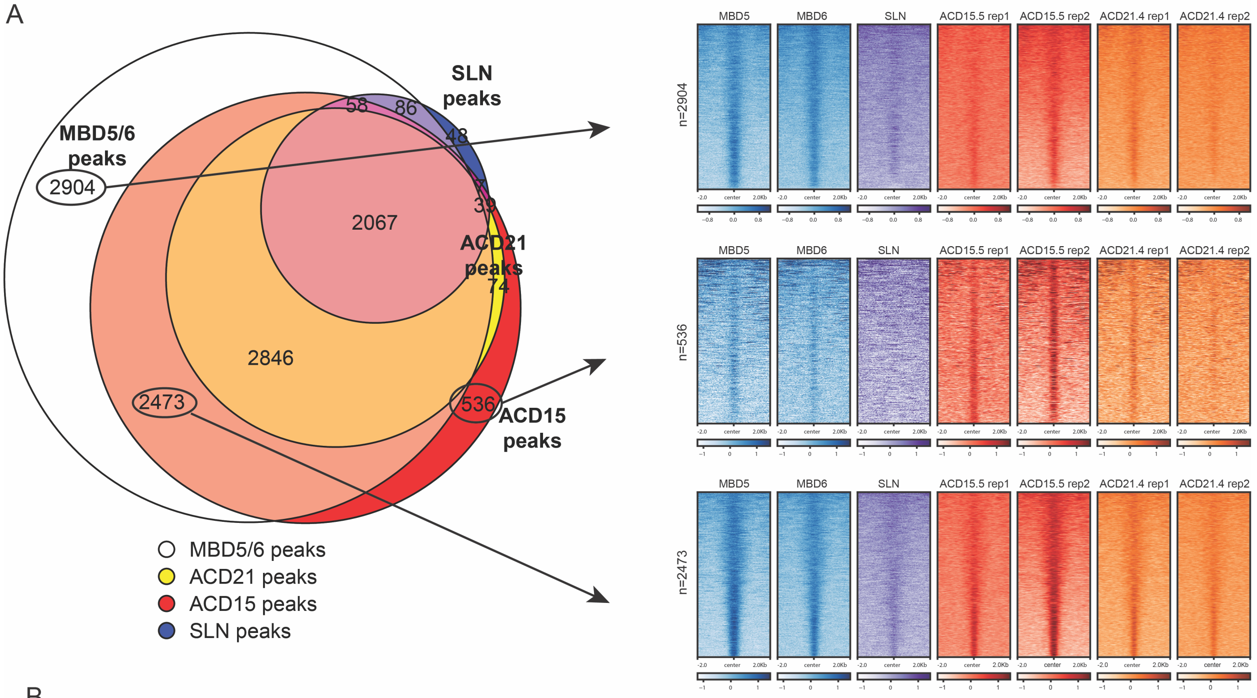

B

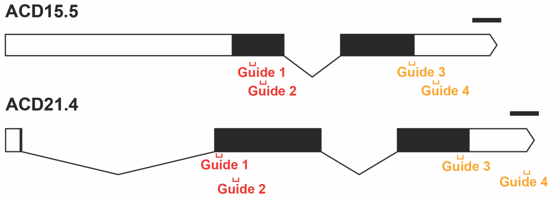

	ACD15.5		ACD21.4		background
acd15.5	guide 2 + 4	Insertion at guide 2 causing STOP codon	guide 2 + 4	WT	Col0
acd21.4	guide 1 + 3	WT	guide 1 + 3	single base insertion (A) at guide 1	Col0
acd15.5 acd21.4	guide 1 + 3	single base insertion (A) at guide 1	guide 1 + 3	37bp deletion at guide 1 causing STOP codon	Col0
adc15.5 acd21.4 sln	guide 1 + 3	single base deletion (A) at guide 1	guide 1 + 3	single base insertion (A) at guide 1	*sln* T-DNA: SALK_090484

C

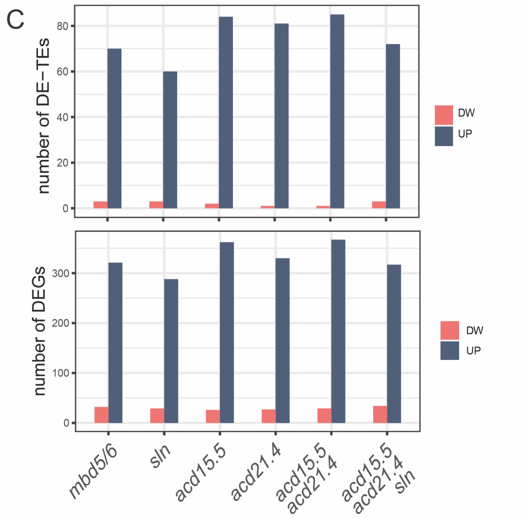

D

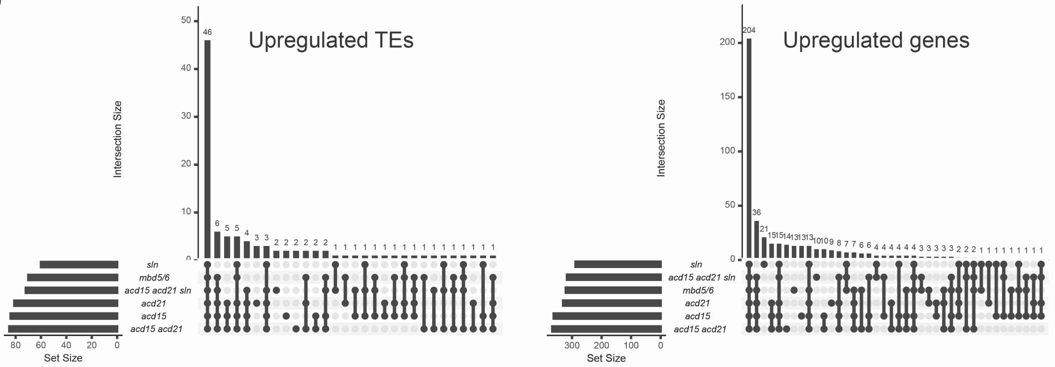

Fig. S1. ChIP-seq and RNA-seq analysis of ACD15 and ACD21.

A) Venn diagram of ChIP-seq peaks showing large overlap between samples. The peak sets indicated with circles (putative MBD5/6 unique, ACD15 unique, or MBD5/6/ACD15 unique peaks) were selected and visualized with heatmaps (right). We noted that at each peaks set groups, enrichment of most proteins was observed, thus suggesting that these regions are bound by all components of the MBD5/6 complex, despite not reaching our stringent significance threshold to be called as peaks. The heatmap shows log2(fold-change) over no-FLAG control. **B)** Scheme of ACD15 and ACD21 genes showing the location of the guide RNAs used for CRISPR/Cas9 mediated mutant generation. The table below shows the mutations obtained in each line. **C)** Bar plots showing the number of differentially expressed TEs (DE-TEs) or differentially expressed genes (DEGs) in the indicated genotypes. **D)** Upset plots showing the intersection of the upregulated genes or TEs found for each genotype. The largest intersection group constitutes loci upregulated in all six mutant lines.

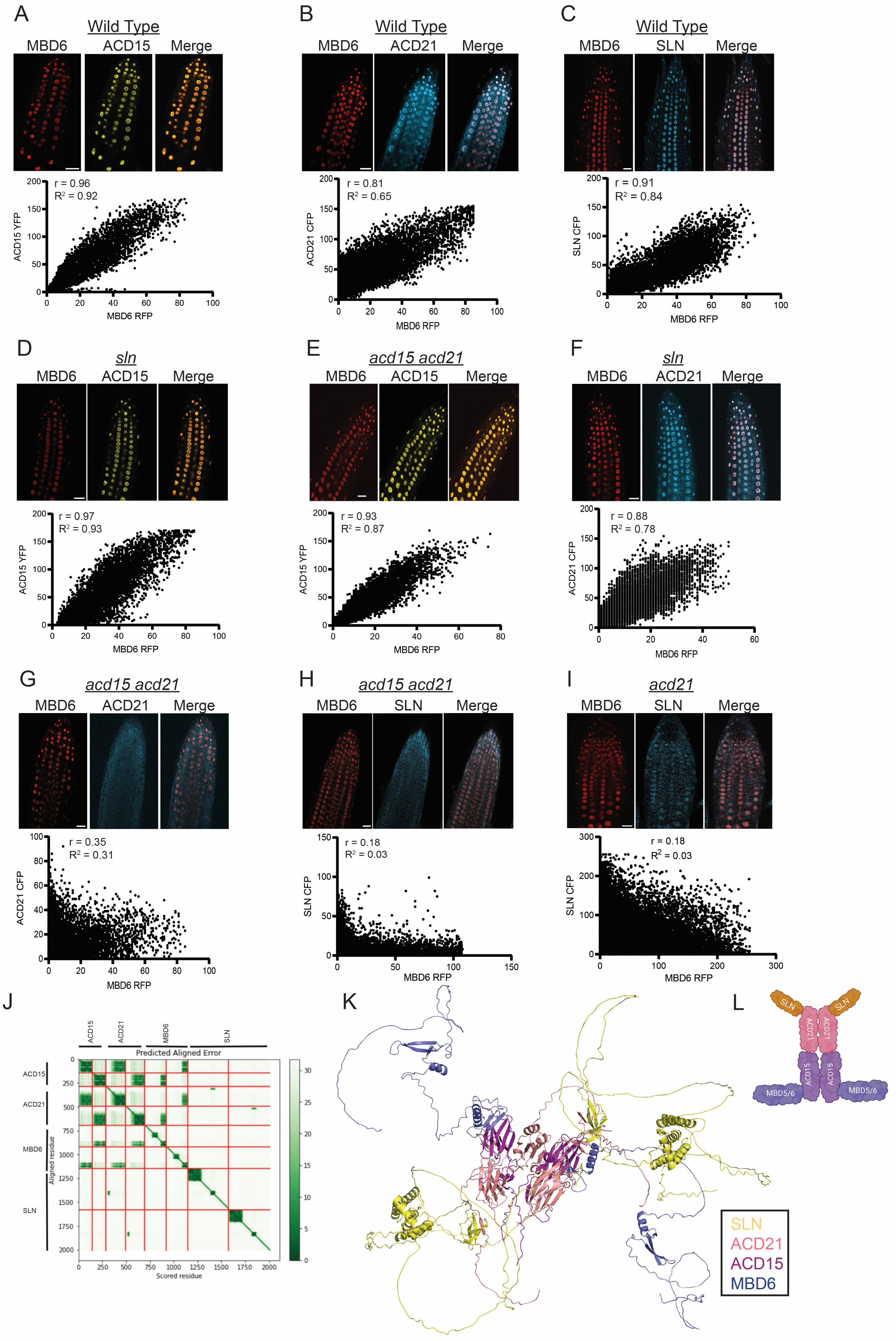

A
Wild Type
MBD6 ACD15 Merge
r = 0.96
R² = 0.92
ACD15 YFP
MBD6 RFP
B
Wild Type
MBD6 ACD21 Merge
r = 0.81
R² = 0.65
ACD21 CFP
MBD6 RFP
C
Wild Type
MBD6 SLN Merge
r = 0.91
R² = 0.84
SLN CFP
MBD6 RFP
D
sln
MBD6 ACD15 Merge
r = 0.97
R² = 0.93
ACD15 YFP
MBD6 RFP
E
acd15 acd21
MBD6 ACD15 Merge
r = 0.93
R² = 0.87
ACD15 YFP
MBD6 RFP
F
sln
MBD6 ACD21 Merge
r = 0.88
R² = 0.78
ACD21 CFP
MBD6 RFP
G
acd15 acd21
MBD6 ACD21 Merge
r = 0.35
R² = 0.31
ACD21 CFP
MBD6 RFP
H
acd15 acd21
MBD6 SLN Merge
r = 0.18
R² = 0.03
SLN CFP
MBD6 RFP
I
acd21
MBD6 SLN Merge
r = 0.18
R² = 0.03
SLN CFP
MBD6 RFP
J
ACD15 ACD21 MBD6 SLN
Predicted Aligned Error
Aligned residue
Scored residue
K
SLN
ACD21
ACD15
MBD6
L
SLN SLN
ACD21 ACD21
ACD15 ACD15
MBD5/6 MBD5/6

Fig. S2. Organization of the MBD5/6 complex structure.
(A-I) Correlation between MBD6-RFP signal and either ACD15-YFP, ACD21-CFP, or SLN-CFP signal in the indicated mutant backgrounds (underlined). Images represent individual z-stack slices of roots from plants co-expressing MBD6 with either ACD15, ACD21, and SLN. Scatter plots indicate signal intensity for each fluorescent protein at each pixel of the image shown. Correlation coefficient: Pearson. Scale bars = 20 μM. **(J-K)** AlphaFold Multimer predicted structure of MBD5/6 complex with two copies each of MBD6, ACDC15, ACD21, and SLN along with confidence score map of the predicted complex. **(L)** Cartoon representation of the core dimeric MBD5/6 complex based on the AlphaFold Multimer prediction. The figure was created with Biorender.com.

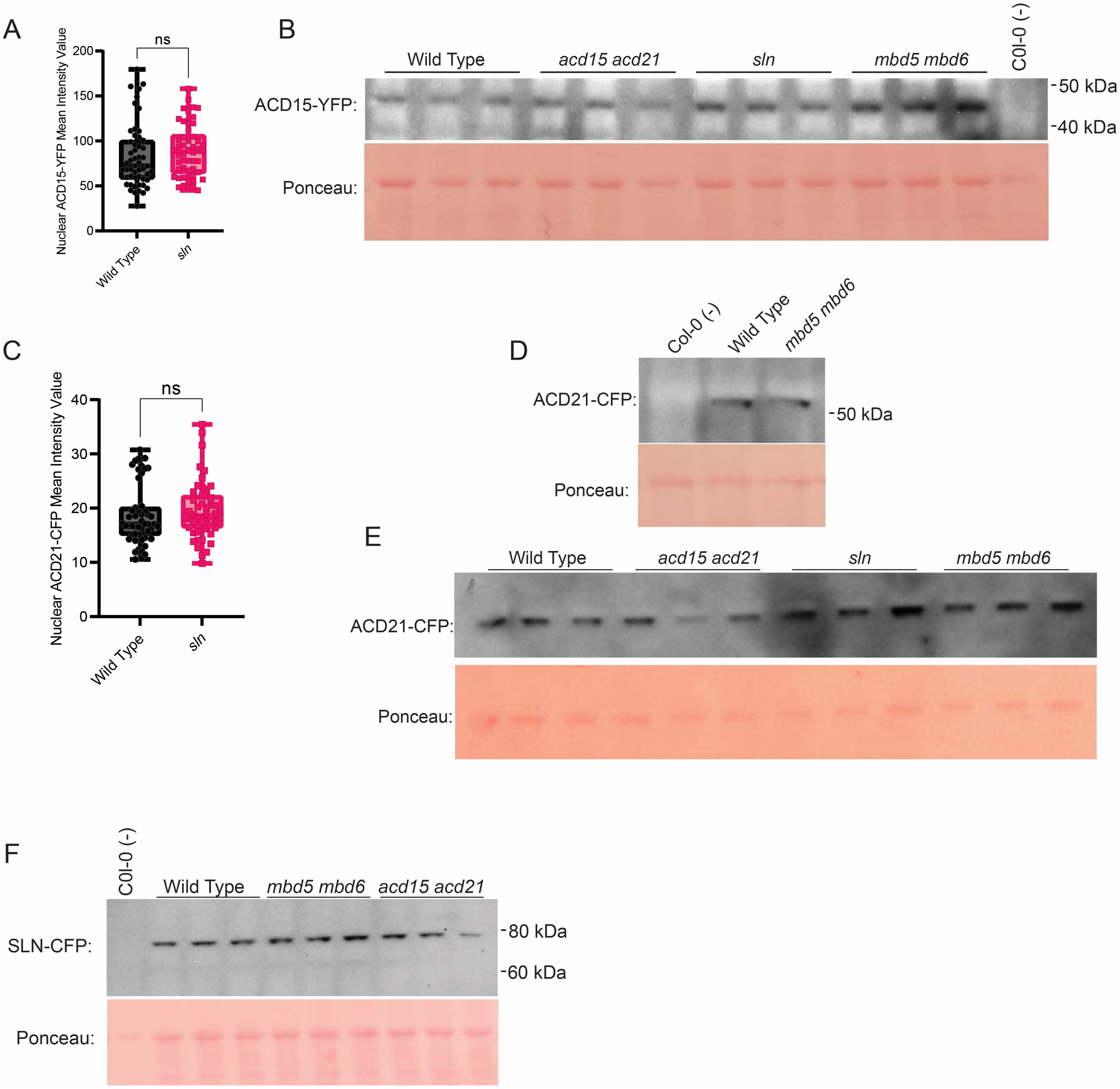

Fig. S3. ACD15, ACD21, and SLN protein levels across plant lines.
(A) ACD15-YFP nuclear intensity in wild-type vs *sln* mutant plants. Comparisons were made using two-tailed t tests (NS: P>=0.05, N=52 and 50 respectively). **(B)** Western blot analysis of ACD15-YFP protein levels along with ponceau stain as a loading control. **(C)** ACD21-CFP nuclear intensity in wild type vs *sln* mutant plants. Comparisons were made using two-tailed t tests (NS: P>=0.05, N=50 per genotype). **(D-E)** Western blot analysis of ACD21-CFP protein levels along with ponceau stains as loading controls. **(F)** Western blot analysis of SLN-CFP protein levels along with ponceau staining as a loading control.

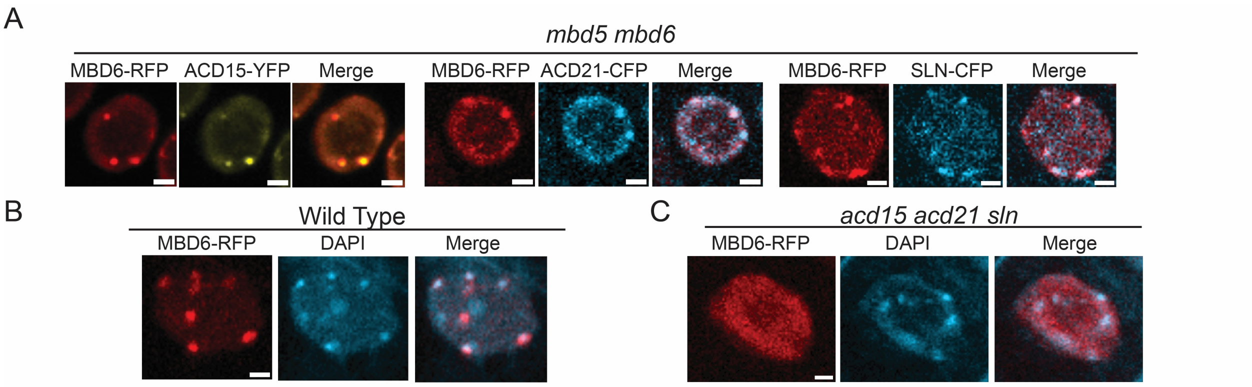

D

	FRAP Recovery Half Time ($t_{1/2}$)		Maximum Plateau Post Bleaching	
	**Wild Type**	***sln***	**Wild Type**	***sln***
MBD6	3.60 (3.175 to 3.991)	2.44 (1.813 to 3.174)	0.93 (0.9137 to 0.9561)	0.61 (0.5826 to 0.6336)
ACD15	3.63 (3.243 to 4.046)	4.93 (4.054 to 5.951)	0.94 (0.9233 to 0.9635)	0.59 (0.5676 to 0.6212)
ACD21	4.30 (3.735 to 4.927)	3.26 (2.448 to 4.220)	0.91 (0.8813 to 0.9351)	0.63 (0.6009 to 0.6625)

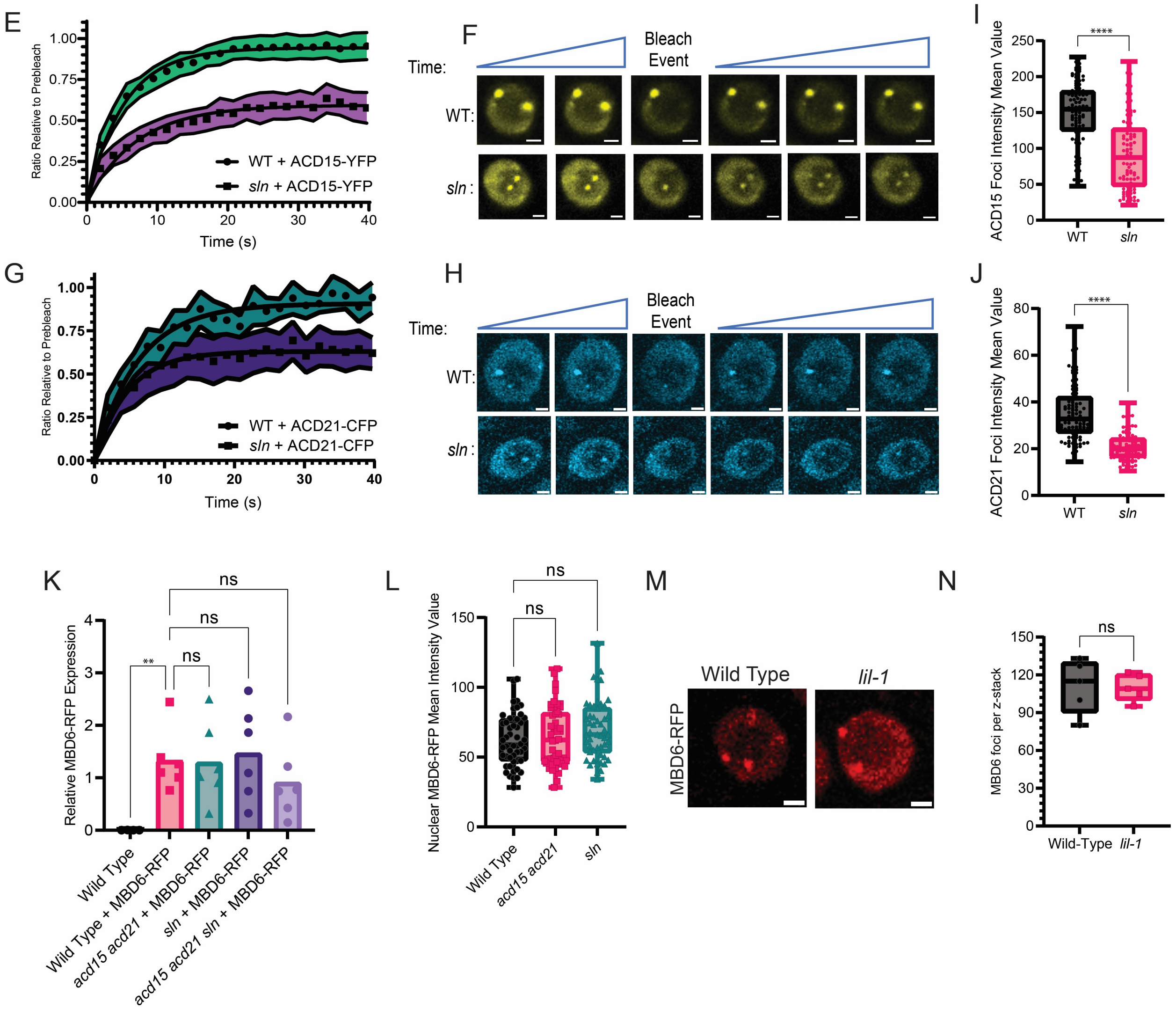

Fig. S4. SLN regulates the nuclear mobility of MBD5/6 complex members.

(A) Representative root nuclei images demonstrating MBD6-RFP overlap with ACD15-YFP, ACD21-CFP, and SLN-CFP. Scale bar = 2μM. **(B-C)** MBD6-RFP signal within DAPI-stained nuclei. Scale bar = 2μM. **(D)** Table of extrapolated values from FRAP curve data fitted with one-phase association liner regression using GraphPad Prism. **(E-H)** FRAP curves of ACD15 and ACD21 along with representative nuclei images of FRAP experiments. Shaded area: 95% confidence interval of FRAP data (N=25 from 5 plants lines), dots: mean values, line: fitted one-phase, non-linear regression. Scale bars = 2μM. **(I-J)** Intensity of ACD15 (I) and ACD21 (J) signal at 100 individual foci from multiple nuclei and plant lines. Comparisons were made using two-tailed t tests (****: P<0.0001). **(K)** Expression of MBD6-RFP transgene across plant lines as measured by RT-qPCR. Comparisons were made using Welch's ANOVA and Dunnet's T3 multiple comparisons test (**: P<0.01, NS: P>=0.05, N=4 for wild type and 5 per mutant genotype). **(L)** MBD6-RFP nuclear intensity across wild type, *acd15 acd21*, and *sln* mutant plant lines. (NS: P>=0.05, N=50 per genotype) **(M)** Representative nuclei showing MBD6-RFP foci in lil-1 mutant and control plants. Scale bars = 2μM. **(N)** MBD6 foci counts across 50 slice Z-stacks of root meristem from five plant lines per genotype. Two Tailed T-test (NS: P>=0.05, N=5 per genotype).

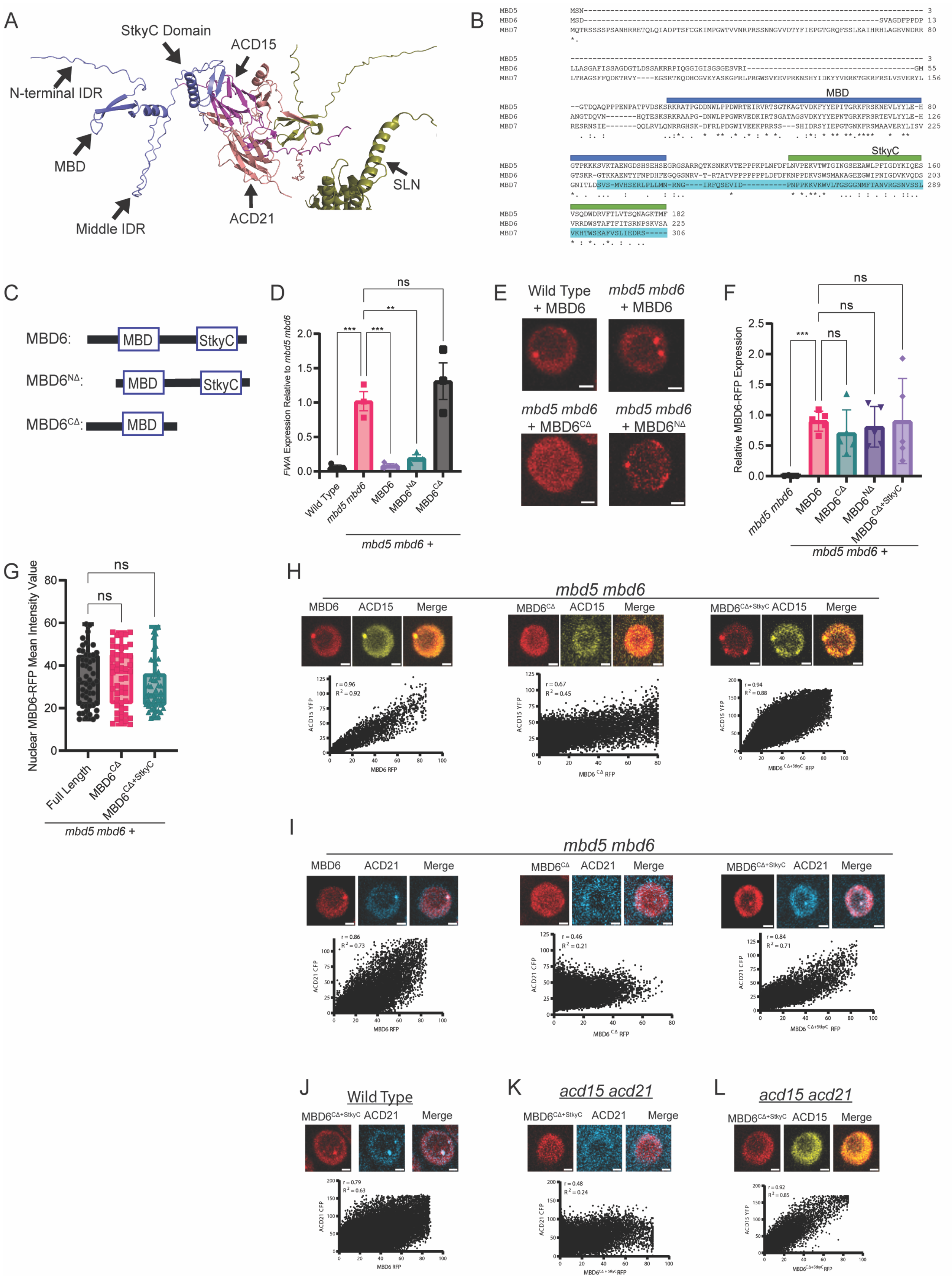
A
StkyC Domain
ACD15
N-terminal IDR
MBD
SLN
ACD21
Middle IDR
B
MBD5
MBD6
MBD7
MBD
StkyC
C
MBD6:
MBD
StkyC
MBD6NΔ:
MBD6CΔ:
D
FWA Expression Relative to mbd5 mbd6
Wild Type
mbd5 mbd6
MBD6
MBD6NΔ
MBD6CΔ
mbd5 mbd6 +
ns
**
***
E
Wild Type + MBD6
mbd5 mbd6 + MBD6
mbd5 mbd6 + MBD6CΔ
mbd5 mbd6 + MBD6NΔ
F
Relative MBD6-RFP Expression
mbd5 mbd6
MBD6
MBD6CΔ
MBD6NΔ
MBD6CΔ+StkyC
mbd5 mbd6 +
G
Nuclear MBD6-RFP Mean Intensity Value
Full Length
MBD6CΔ
MBD6CΔ+StkyC
mbd5 mbd6 +
H
mbd5 mbd6
MBD6
ACD15
Merge
MBD6CΔ
MBD6CΔ+StkyC
r = 0.96
R2 = 0.92
r = 0.67
R2 = 0.45
r = 0.94
R2 = 0.88
ACD15 YFP
MBD6 RFP
I
mbd5 mbd6
ACD21
r = 0.86
R2 = 0.73
r = 0.46
R2 = 0.21
r = 0.84
R2 = 0.71
ACD21 CFP
J
Wild Type
r = 0.79
R2 = 0.63
K
acd15 acd21
r = 0.48
R2 = 0.24
L
acd15 acd21
r = 0.92
R2 = 0.85

Fig. S5. The StkyC domain is required for MBD6's function.
(A) Protein structure representation of AlphaFold Multimer prediction of MBD6 with ACD15. Domains of MBD6 are annotated. **(B)** Protein alignment of MBD5, MBD6, and MBD7. MBDs and StkyC regions of MBD5, MBD6, and MBD7 are labelled. **(C)** Graphical representation of MBD6 deletion mutants. **(D-E)** RT-qPCR of *FWA* expression comparing *mbd5 mbd6* plants expressing MBD6 deletion mutants (D), along with representative images of MBD6 deletion mutants in root nuclei (E). Comparisons made using Brown-Forsythe ANOVA with Dunnett's multiple comparisons test (***: P<0.001, **: P<0.01, NS: P>=0.05, N=3 per genotype. Scale bars = 2µM). **(F)** RT-qPCR comparison of MBD6 deletion mutant expression compared to full length MBD6-RFP. Comparisons were made using Welch's ANOVA and Dunnet's T3 multiple comparisons test (***: P<0.001, NS: P>=0.05, N=4 for wild type and 5 per MBD6 allele). **(G)** MBD6-RFP nuclear intensity of MBD6 deletion mutants in *mbd5 mbd6* mutant plant lines. Comparisons made using Kruskal-Wallis test with Dunn's multiple comparisons test (NS: P>=0.05, N=50 per MBD6 allele). **(H-L)** Correlation of MBD6-RFP domain, deletion mutants with ACD15-YFP or ACD21-CFP along with representative nuclear images. Correlation coefficient: Pearson. Scale bars = 2µM.

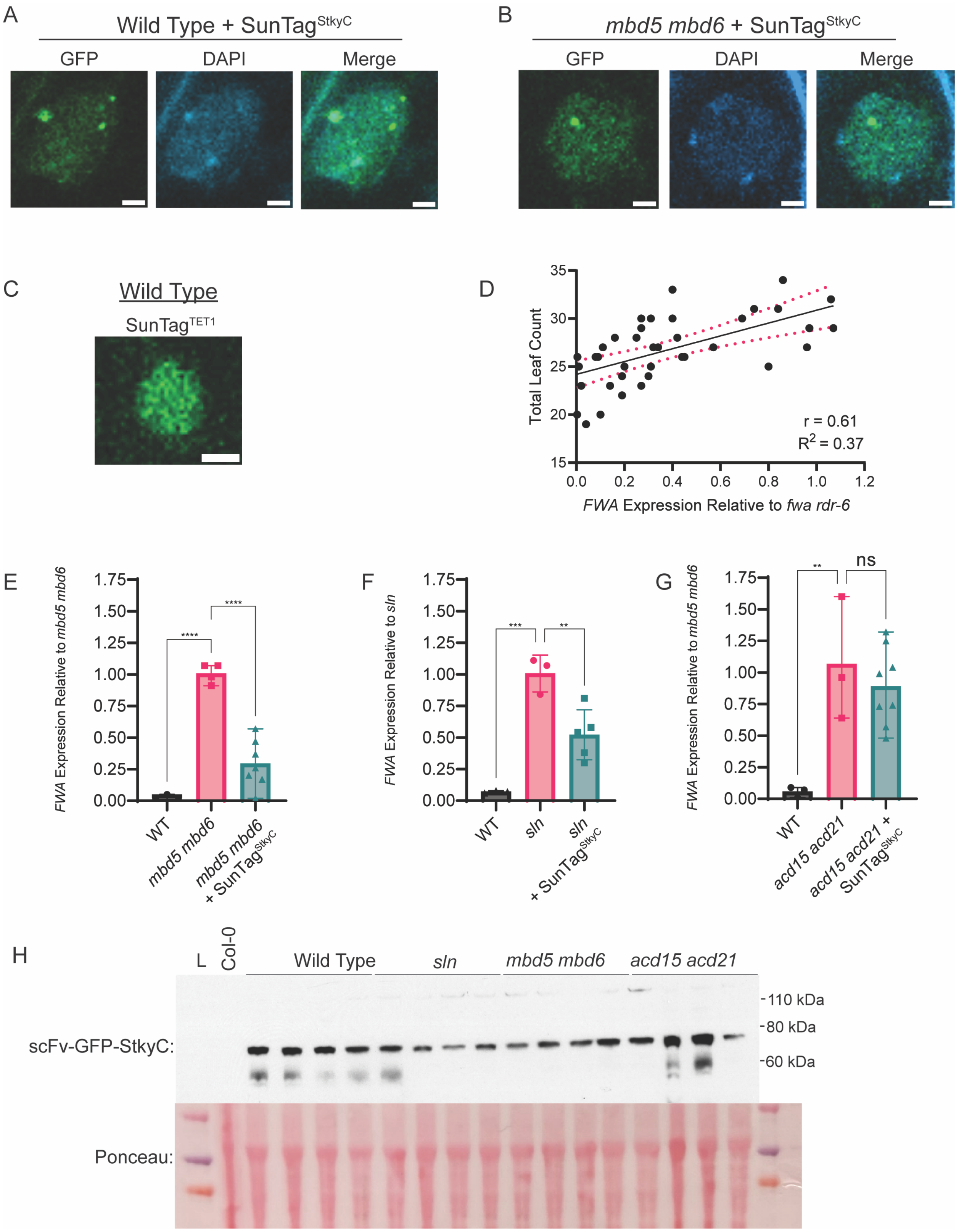
A
Wild Type + SunTagStkyC
GFP
DAPI
Merge
B
mbd5 mbd6 + SunTagStkyC
GFP
DAPI
Merge
C
Wild Type
SunTagTET1
D
Total Leaf Count
FWA Expression Relative to fwa rdr-6
r = 0.61
R2 = 0.37
E
FWA Expression Relative to mbd5 mbd6
WT
mbd5 mbd6
mbd5 mbd6 + SunTagStkyC
F
FWA Expression Relative to sln
WT
sln
sln + SunTagStkyC
G
FWA Expression Relative to mbd5 mbd6
ns
WT
acd15 acd21
acd15 acd21 + SunTagStkyC
H
L
Col-0
Wild Type
sln
mbd5 mbd6
acd15 acd21
110 kDa
80 kDa
60 kDa
scFv-GFP-StkyC:
Ponceau:

Fig. S6. SunTag^{StkyC} drives the formation of MBD5/6 nuclear foci.
(A-B) SunTag^{StkyC} expressing root nuclei stained with DAPI. Scale bars = 2μM. **(C)** Representative image of a root nucleus expressing SunTag^{TET1}. Scale bar = 2μM. **(D)** Correlation of SunTag^{StkyC} *FWA* expression with leaf counts of individual T1 plants from Figure 5E-F. Correlation coefficient: Pearson. **(E-G)** RT-qPCR of *FWA* in *mbd5 mbd6* (N=3, 4, and 7 respectively), *sln* (N=3, 4, and 10 respectively), and *acd15 acd21* (N=3, 3, and 8 respectively) plants with and without SunTag^{StkyC}. Comparisons made using Brown-Forsythe ANOVA with Dunnett's multiple comparison test for each qPCR experiment (****: P<0.0001, ***: P<0.001, **: P<0.01, *: P<0.05, NS: P>=0.05). **(H)** Western blot analysis of scFv-GFP-StkyC using HA antibody along with ponceau staining as a loading control.

Table S1. (Separate File)
Table showing merged IP-MS experiments. The numbers reported correspond to MS/MS counts. Col0 samples are no-FLAG negative controls. Each independent experiment is annotated with a shaded color.

Protein	AtG ID
MBD6	AT5G59380.1
MBD5	AT3G46580.1
ACD15	AT1G76440.1
ACD21	AT1G54850.1
SLN	AT5G37380.1

Table S2. Gene identifiers for MBD5/6 complex.
All members of the MBD5/6 complex are listed along with gene identifiers associated with each gene for online *A. thaliana* database (https://www.arabidopsis.org/).

Primer name	Sequence
FWA RT-qPCR Fw	TTAGATCCAAAGGAGTATCAAAG
FWA RT-qPCR Rev	CTTTGGTACCAGCGGAGA
IPP2 RT-qPCR Fw	GTATGAGTTGCTTCTCCAGCAAAG
IPP2 RT-qPCR Rev	GAGGATGGCTGCAACAAGTGT
RFP-qPCR Fw	ATGGCCTCCTCCGAGGACGTCATCA
RFP-qPCR Rev	CTTGGTCACCTTCAGCTTGGCGGTC

Table S3. qPCR primer sequences.
List of qPCR primers, forward (Fw) and reverse (Rev), along with DNA sequences (5'-3').
